# An observational study on surgically treated adult idiopathic scoliosis patients’ quality of life outcomes at 1- and 2-year follow-ups and comparison to controls

**DOI:** 10.1186/s13013-017-0118-y

**Published:** 2017-04-12

**Authors:** Jennifer C. Theis, Anna Grauers, Elias Diarbakerli, Panayiotis Savvides, Allan Abbott, Paul Gerdhem

**Affiliations:** 1 Faculty of Health Science and Medicine, Bond Institute of Health and Sport, Bond University, 2 Promethean Way, Robina, Queensland 4226 Australia; 2 Department of Orthopaedics, Department of Clinical Science, Intervention and Technology (CLINTEC), Karolinska Institutet, Karolinska University Hospital, SE-141 86 Stockholm, Sweden; 3 Department of Orthopaedics, Sundsvall and Härnösand County Hospital, 85186 Sundsvall, Sweden; 4 Department of Physical Therapy, Karolinska University Hospital, SE-141 86 Stockholm, Sweden; 5 Division of Physiotherapy, Department of Neurobiology, Care Sciences and Society, Karolinska Institutet, SE-141 86 Stockholm, Sweden; 6 Department of Medical and Health Sciences, Division of Physiotherapy, Faculty of Health Sciences, Linköping University, SE-58183 Linköping, Sweden

**Keywords:** Scoliosis, Adults, Idiopathic, Surgery

## Abstract

**Background:**

Prospective data on health-related quality of life in patients with idiopathic scoliosis treated surgically as adults is needed. We compared preoperative and 1- and 2-year follow-up data in surgically treated adults with idiopathic scoliosis with juvenile or adolescent onset. Results were compared to untreated adults with scoliosis and population normative data.

**Methods:**

A comparison of preoperative and 1- and 2-year follow-up data of 75 adults surgically treated for idiopathic scoliosis at a mean age of 28 years (range 18 to 69) from a prospective national register study, as well as a comparison with age- and sex-matched data from 75 untreated adults with less severe scoliosis and 75 adults without scoliosis, was made. Outcome measures were EuroQol-5 dimensions (EQ-5D) and Scoliosis Research Society (SRS)-22r questionnaire.

**Results:**

In the surgically treated, EQ-5D and SRS-22r scores had statistically significant improvements at both 1- and 2-year follow-ups (all *p* < 0.015). The effect size of surgery on EQ-5D at 1-year follow-up was large (*r* = −0.54) and small-medium (*r* = −0.20) at 2-year follow-up. The effect size of surgery on SRS-22r outcomes was medium-large at 1- and 2-year follow-ups (*r* = −0.43 and *r* = −0.42 respectively). At the 2-year follow-up, the EQ-5D score and the SRS-22r subscore were similar to the untreated scoliosis group (*p* = 0.56 and *p* = 0.91 respectively), but lower than those in the adults without scoliosis (*p* < 0.001 for both comparisons).

**Conclusions:**

Adults with idiopathic scoliosis experience an increase in health-related quality of life following surgery at 2-year follow-up, approaching the health-related quality of life of untreated individuals with less severe scoliosis, but remain lower than normative population data.

## Background

Scoliosis in adults has two primary etiologies, degenerative scoliosis and idiopathic scoliosis, defined as a skeletal deformity with a coronal plane Cobb angle greater than 10° in the skeletally mature individual [1, 2]. Adult idiopathic scoliosis stems from a progression of adolescent or childhood idiopathic scoliosis often associated with secondary spinal degeneration [1, 2]. Regardless of pathogenesis, adult scoliosis is associated with back pain, radicular pain, claudication symptoms, and continued degenerative changes [1, 2].

Considering the suggested impact of scoliosis on health-related quality of life (HRQOL) [3, 4], in addition to correcting the deformity and halting curve progression, interventions for adult scoliosis should attempt to relieve pain, improve function, and thus improve patient HRQOL. Additionally, radiographic changes have been suggested to poorly correlate with HRQOL outcome measures for adults with scoliosis [3]. For this reason, both generic patient reported HRQOL instruments like the EuroQol-5 dimensions (EQ-5D) and the preferred disease-specific outcome measure, Scoliosis Research Society (SRS)-22r, are suggested to evaluate treatment effectiveness in this population [5].

Even though evidence for effectiveness of non-operative treatment are scarce, such attempts should be performed before surgical procedures [6–8]. Patients with less deformity are more likely to benefit from non-operative treatment [7, 9]. Studies suggest surgery has a positive impact on deformity and HRQOL in adults with scoliosis [6, 7, 10–13]. However, few studies with prospective designs reporting HRQOL pre- and postoperatively for adults with scoliosis exist in the literature [6, 7, 10–12]. In addition, few report data specifically for adults with idiopathic scoliosis [14].

The aim of this prospective study is to contribute to the research pertaining to surgically treated adults with idiopathic scoliosis by utilizing the Swedish Spine register (Swespine) to (1) assess the effect of surgery on patient’s HRQOL comparing preoperative and 1- and 2-year follow-up EQ-5D and SRS-22r scores, (2) compare 2-year follow-up EQ-5D and SRS-22r scores for adults with idiopathic scoliosis to age- and sex-matched controls of untreated individuals with less severe scoliosis as well as individuals without scoliosis.

## Methods

### Participants

This is an analysis of prospectively collected data from the Swedish Spine register (Swespine). Surgical procedures for spinal deformity, such as scoliosis, have been included in the register since 2006 [15]. Surgeon reported variables included data on surgical procedures and complications. Incidences of reoperation were registered by the surgeon responsible for the procedure.

Patient-reported HRQOL outcome measures including the EQ-5D and, since 2008, the SRS-22r. Patients completed the questionnaires preoperatively and again via mail at 1 and 2 years. Patient-reported complications are gathered at 1-year follow-up and defined as thrombosis, pulmonary embolism, and infection treated with antibiotics within the first three months following surgery.

The EQ-5D contains a question in five dimensions including mobility, self-care, usual activities, pain/discomfort, and anxiety/depression, scored from 1 (no problem) to 3 (extreme problems) that is converted to an index score between 1 (perfect health) and −0.59 (a health state worse than death) representing the societal view of health [16].

The disease-specific SRS-22r contains 22 questions covering five domains: function/activity, pain, self-perceived image, mental health, and satisfaction with treatment, scored from 1 (worst) to 5 (best) [17]. The 20 questions related to the first four domains were analyzed in this study and collated to make the SRS-22r subscore.

**Fig. 1:**
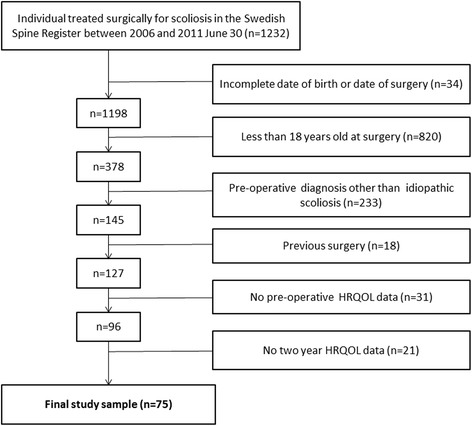
Flow chart for the inclusion of participants from the Swedish Spine Register, HRQOL—health-related quality of life measured by EuroQol-5D and Scoliosis Research Society Questionnaire-22r

Inclusion criteria for this study was (1) surgery prior to June 30, 2011, (2) age ≥18 years at time of surgery, (3) primary diagnosis of idiopathic scoliosis with juvenile or adolescent onset, (4) preoperative and 2-year follow-up data reported for EQ-5D and/or SRS-22r, and (5) no prior surgical intervention. The participant extraction from the Swespine register is outlined in Fig. 1. The final study cohort included 75 patients.

### Controls

Controls were matched at a 1:1 ratio for (i) sex and (ii) age corresponding to the 2-year follow-up for the cases. We chose to use two control populations: untreated individuals with scoliosis and individuals without scoliosis.

Data on previously observed, untreated, individuals with juvenile or adolescent idiopathic scoliosis collected are continuously collected by our research group, parts of which have been presented earlier [18]. These individuals were asked to fill out the EQ-5D and SRS-22r questionnaires, and 75 controls were selected out of 347 available individuals with EQ-5D and SRS-22r data.

The Swedish population register was used to identify individuals without scoliosis, and the EQ-5D and SRS-22r questionnaires were mailed to 407 randomly selected adults (aged 18–70) with 229 respondents, from which we selected 75 individuals. These were not physically examined. Data from this cohort have been presented elsewhere [18, 19].

### Radiology

Preoperative radiological images were retrieved for all 75 patients, and postoperative images for 72 out of 75 patients and classified by two of the authors according to Cobb [2] and Lenke et al. [20].

### Statistics

The power calculation was based on the postoperative effect sizes observed in adults with scoliosis [13]. The priori sample size was calculated using G*Power 3.1.7 (Universität Kiel, Germany) [21] with a Wilcoxon signed-rank test, effect size of 0.8, type I error *α* = 0.05, and power = 0.8, yielding a required sample size of 12. Due to non-parametric distributions, effect size was calculated using *r* = *z*/√*N* (*z* = *z* value, *N* = total number of observations used to calculate *z*).

The Wilcoxon signed-rank test was used for paired samples, the Welch-Satterthwaite *t* test for unpaired samples, and the Pearson chi-square test for differences in the different EQ-5D domains. To analyze age differences, the patients were divided into two age groups: ≥25 and <25 years. In case of missing data, cases were excluded analysis by analysis. Descriptive statistics were reported as mean (SD), median (25th, 75th percentile), or number (%). A Bonferroni adjustment of significance was made from *p* ≤ 0.05 to *p* ≤ 0.017. IBM SPSS Statistics version 20 (Armonk, NY, 2011) was used for statistical analysis.

### Non-response analysis

The 31 participants without preoperative HRQOL data were not significantly different regarding age at surgery (*p* = 0.56), primary Cobb angle (*p* = 0.26), or number of fused vertebrae (*p* = 0.03) than the 96 individuals with preoperative HRQOL data (Fig. 1). The 21 participants lost to 2-year follow-up were not significantly different regarding preoperative age at time of surgery (*p* = 0.99), primary Cobb angle (*p* = 0.50), number of fused vertebrae (*p* = 0.55), EQ-5D index value (*p* = 0.18), or SRS-22r subscore (*p* = 0.15) to the 75 participants with 2-year follow-up data (Fig. 1). One patient died prior to 2-year follow-up. This death was not linked to the surgical event.

## Results

In each group of 75 individuals, there were 60 (80%) females. In the group of patients, mean age was 27 (18–69) years at the time of surgery. Of these, 54 patients were aged <25 years (mean age 20) and 21 were >25 years (mean age 46). Preoperative Cobb angle was 54 (8) degrees and postoperative 25 (10) degrees. Other descriptive data for the patients are outlined in Table 1. Regarding the controls, the mean age of the untreated individuals with scoliosis was 30 (18–67) years, and their last available radiograph showed a mean Cobb angle of 28 (14) degrees. The mean age of the individuals without scoliosis was 32 (17–69) years.

**Table 1:** Surgical, complication, and reoperation characteristics of study participants as reported in the Swedish Spine Registry. Complications reported by participants at 1-year follow-up; all other data reported by surgeon at time of initial surgery or reoperation. Data is shown as mean (SD) or number. *n* = 75 except where otherwise indicated

Characteristic	
Lenke classification (*n* = 70)	
Type 1	23
Type 2	7
Type 3	10
Type 4	2
Type 5	15
Type 6	13
Procedure type, number of patients	
Anterior	6
Posterior	68
Combined	1
Levels fused, number (*n* = 74)	11 (3)
Fusion to sacrum, number (*n* = 74)	4
Intraoperative blood loss, L (*n* = 71)	1.5 (1.0)
Hospital stay from operation to discharge, days (*n* = 73)	8 (7, 10)
Complications	
Nerve root injury, dural lesion, spinal cord injury, mortality	0
Thrombosis	1
Pulmonary embolism	4
Surgical site infection treated with antibiotics	2
Reoperation, number of participants with reoperation, (indication)	4 (1 implant replacement, 2 surgical site infection spine, 1 infection at bone graft harvest site)

**Fig. 2:**
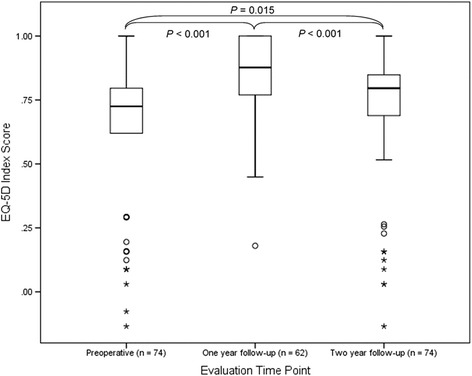
EuroQol-5 dimension (EQ-5D) questionnaire index scores, *boxes* represent inner quartile range (IQR) with median denoted by *horizontal line*, *inner fences* represent minimum and maximum values or 1.5 times IQR, *circles* outlier between 1.5 and 3 time IQR, *asterisks* far outlier greater than three times IQR. There was significant improvement in EQ-5D index scores from preoperative to 1- and 2-year follow-ups, with a significant decrease in index scores from 1- to 2-year follow-ups

There was a statistically significant improvement in EQ-5D index scores from preoperative to 1-year follow-up with 79% of respondent’s scores improving while from preoperative to 2-year follow-up 53% of reported scores improved (Fig. 2). However, there was a statistically significant decrease in EQ-5D index scores from 1- to 2-year follow-up (Fig. 2). The effect size of surgery on 1-year follow-up EQ-5D index scores was large (*r* = −0.54) while at 2-year follow-up the effect was small towards medium (*r* = −0.20) [22]. The 2-year follow-up EQ-5D index was similar to the untreated individuals with scoliosis but continued to have statistically significant worse scores than the individuals without scoliosis (Table 2).

**Table 2:** EuroQol-5D-3L (EQ-5D) dimension and index scores for adult idiopathic scoliosis cohort at 2-year follow-up compared to untreated scoliosis patients and population matched scores. Percentage of participants reporting level of problem per dimension where level 1 indicates no problem, level 2 indicates some problems, and level 3 indicates extreme problems. UK Index reported as mean (SD)

EQ-5D		Number (%) of participants reporting level of problem			p*	p**
		2-year follow-up *n* = 74	Untreated patients *n* = 75	Matched normative *n* = 75		
Mobility	Level 1	64 (86%)	69 (92%)	72 (96%)	0.28	0.04
	Level 2	10 (14%)	6 (8%)	3 (4%)		
	Level 3	0	0	0		
Self-care	Level 1	70 (96%)	73 (97%)	75 (100%)	0.63	0.08
	Level 2	3 (4%)	2 (3%)	0		
	Level 3	0	0	0		
Usual activities	Level 1	48 (66%)	63 (84%)	73 (97%)	0.019	<0.001
	Level 2	22 (30%)	12 (16%)	2 (3%)		
	Level 3	3 (4%)	0	0		
Pain/discomfort	Level 1	23 (31%)	22 (29%)	52 (69%)	0.003	<0.001
	Level 2	41 (55%)	53 (71%)	22 (29%)		
	Level 3	10 (14%)	0	1 (1%)		
Anxiety/depression	Level 1	45 (61%)	44 (59%)	54 (72%)	0.32	0.33
	Level 2	27 (36%)	31 (41%)	19 (25%)		
	Level 3	2 (3%)	0	2 (3%)		
EQ-5D index		0.72 (0.28)	0.74 (0.25)	0.88 (0.16)	0.56	<0.001

*p value for the comparison between surgically treated and untreated patients

**p value for the comparison between surgically treated patients and matched normative data

**Fig. 3:**
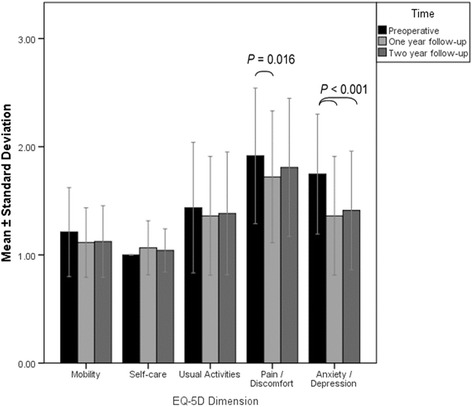
Preoperative and 1- and 2-year follow-up EQ-5D dimension scores, mean ± standard deviation. Significant difference found for pain/discomfort dimension between preoperative and 1-year follow-up and from preoperative to 1- and 2-year follow-ups for anxiety/depression dimension, significance levels indicated

EQ-5D dimension scores for pain/discomfort and anxiety/depression demonstrated statistically significant improvement between preoperative and follow-up time points, with no significant differences noted for any other dimension between follow-ups (Fig. 3). At the 2-year follow-up, the EQ-5D dimension of pain/discomfort was significantly lower than that in both control groups, and the domain usual activities was lower than in individuals without scoliosis (Table 2).

**Fig. 4:**
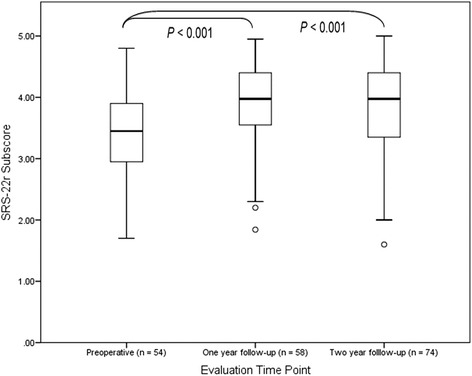
Scoliosis Research Society-22r (SRS-22r) questionnaire subscores, *boxes* represent inner quartile range (IQR) with median denoted by *horizontal line*, *inner fences* represent minimum and maximum values or 1.5 times IQR, *circles* outlier between 1.5 and 3 time IQR, *astersisks* far outlier greater than three times IQR. There was a significant increase in SRS-22r subscores from preoperative to 1- and 2-year follow-ups with no significant difference between follow-up scores

SRS-22r subscores had statistically significant improvement between preoperative and follow-up time points with no significant difference between follow-ups (Fig. 4). At 1- and 2-year follow-ups, 79 and 78% of participant’s SRS-22r subscores improved compared to those of preoperative results. The overall medium to large (*r* = −0.43 and *r* = −0.42 respectively) effect of surgery on SRS-22r outcomes remained relatively constant from 1- to 2-year follow-ups. The over 25 years age group had significantly lower SRS-22r subscores preoperatively and at both 1- and 2-year follow-ups compared to the younger age group (Table 3), with corresponding results for the EQ-5D index (data not shown).

**Table 3:** Scoliosis Research Society (SRS)-22r questionnaire domain and subscore at 2-year follow-up for adult idiopathic scoliosis participants 25 years or older compared to those under 25 years. Data shown as mean (SD)

SRS-22r domain	At least 25 years old (*n* = 21)	Under 25 years old (*n* = 54)	*p*
Function	3.6 (0.8)	4.3 (0.8)	0.001
Pain	3.3 (1.2)	4.0 (1.0)	0.020
Image	3.5 (0.7)	3.9 (0.9)	0.052
Mental health	3.4 (0.9)	3.9 (0.8)	0.037
SRS subscore	3.4 (0.8)	4.0 (0.7)	0.006

*n* number of participants

**Fig. 5:**
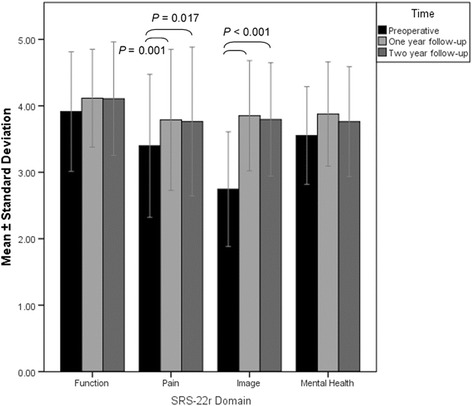
Preoperative and 1- and 2-year follow-up SRS-22r domain scores, mean ± standard deviation. Significant difference found for pain domain between preoperative and 1-year follow-up and image domain between preoperative and both 1- and 2-year follow-ups, significance levels indicated

Statistically significant improvement in the SRS-22r pain domain occurred between preoperative and 1-year follow-up and continued at 2-year follow-up (Fig. 5). A similar statistically significant improvement was seen in the image domain between preoperative and 1- and 2-year follow-up (Fig. 5). At 2-year follow-up, all domains except for the image domain score was significantly lower in the older age group compared to the younger age group (Table 3). When comparing the surgically treated group and the untreated scoliosis group, the SRS-22r subscore and all domains were similar at the 2-year follow-up. However, the SRS-22r subscore and all domain scores, except the mental health domain, continued with statistically significant lower scores than normative population data at 2-year follow-up (Table 4).

**Table 4:** SRS-22r domain scores and subscores shown as mean (SD) for surgically treated patients at 2-year follow-up, age-matched untreated individuals with less severe scoliosis, and age-matched individuals data from individuals without controls

SRS-22r domain	2-year follow-up (*n* = 74)	Untreated individuals with scoliosis (*n* = 75)	Individuals without scoliosis (*n* = 75)	p*	p**
Function	4.1 (0.9)	4.2 (0.8)	4.7 (0.4)	0.25	<0.001
Pain	3.8 (1.1)	3.9 (1.0)	4.6 (0.6)	0.61	<0.001
Image	3.8 (0.9)	3.7 (0.8)	4.5 (0.5)	0.71	0.001
Mental health	3.8 (0.8)	3.7 (0.8)	4.1 (0.8)	0.45	0.018
SRS subscore	3.9 (0.8)	3.9 (0.7)	4.5 (0.5)	0.91	<0.001

*p value for the comparison between surgically treated and untreated patients

**p value for the comparison between surgically treated patients and matched normative data

Seven patients sustained at least one complication or reoperation (Table 1). The SRS-22r subscore and EQ-5D index at 1 and 2 years did not differ when compared to the patients without complications or reoperations (all *p* > 0.19).

## Discussion

This study found an overall significant improvement in HRQOL outcome measures at both 1 and 2-year follow-ups, potentially peaking at 1-year follow-up. HRQOL were similar to untreated scoliosis patients with smaller curves, but outcomes remained lower than population matched scores at 2-year follow-up.

The suggested positive HRQOL impact of surgery for adults with scoliosis is consistent with research summarized in recent reviews [10–13, 23]. In addition, the surgically treated individuals improved their HRQOL to a level comparable to untreated individuals with scoliosis. However, more than one fifth of the patients did not improve their HRQOL at all. Whether these achieve other benefits from surgery, such as better long-term pulmonary function, or later benefits in terms of HRQOL cannot be determined from the current study design.

The overall trend of postoperative HRQOL outcomes appears inconsistent in the current research. One study reported no change in SRS-22, Oswestry Disability Index (ODI), and Short Form-12 from 1 to 2-year follow-ups for surgically treated adult deformity patients [24]. Zimmerman et al. reported that in an adult scoliosis population, SRS-22 domain and subscores continued to significantly increase until 2-year follow-up while ODI and Short Form-36 components changed little after 6 months [25]. The SRS-22r results in our study follow the trend of improvement suggested by Glassman et al. [24] and Yoshida et al [14] with leveling off between 1- and 2-year follow-ups. Whether this is true also at longer follow-ups in adults is not known, necessitating further studies.

Even though the SRS-22r has been reported as the HRQOL outcome of choice in the surgically treated scoliosis population [26], the EQ-5D index is an important general HRQOL outcome measure, especially in discussion of cost utility. However, very few studies exist regarding EQ-5D results in the scoliosis population, especially adult surgically treated scoliosis patients. Burström et al. [16] suggested an EQ-5D index Swedish reference value of 0.89 for individuals 20–29 years old, comparable to this study’s matched value and 1-year follow-up value (both 0.88) and higher than preoperative values (0.73). The suggested reference value is also higher than 2-year follow-up values reported in this study (0.80). In a surgically treated adolescent idiopathic scoliosis group, EQ-5D index values remained unchanged from 1- to 2-year follow-ups (0.82) [15]. Decreasing values were seen in the current study, possibly suggesting skeletal maturity at the time of surgery as a critical surgical consideration in regard to EQ-5D index outcomes. The behavior of specific dimensions and domains of the HRQOL tools highlight surgery as a positive intervention on critical aspects of quality of life including pain, anxiety, and image. Studies comparing non-operative to operative management of adult scoliosis suggests that surgical intervention is more effective on HRQOL outcomes and might be more cost-effective, at least in the short term [10, 23].

Of further consideration is the minimal clinical important difference (MCID). The median difference in SRS-22r was 0.5 from preoperative to 2-year follow-up and higher than the MCID (0.4) suggested by Crawford et al. [27], based on a population with a mean age of 53 years. MCID for EQ-5D has not been determined in the treatment of idiopathic scoliosis.

The discrepancy in the significant decline in the EQ-5D index from 1- to 2-year follow-ups while the SRS-22r subscores remained relatively similar might relate to reported poor concurrent validity between the two instruments found in the adolescent idiopathic scoliosis population [28].

The inclusion of the EQ-5D is a strength of this study as it expands the discussion of patient-reported HRQOL outcome measures applicable to the adult scoliosis population and introduces an index value. However, exclusion of the ODI, a commonly used HRQOL outcome measures in adult scoliosis research, and the availability of the SRS-22r scores only after 2008 are limitations of this study in the context of previous research.

Other limitations include the response rate and the comparably low age of the study group. The response rate is comparable to other studies using register data [29]. In the Norwegian spine register, non-respondents had similar outcome as respondents [30]. During the start of the deformity part of the SweSpine register, some clinics failed to handle pre- and postoperative questionnaires. However, non-response analyses indicated no substantial differences in baseline measures between participants and non-participants. We therefore do not believe the response rate indicate a selection bias.

The small subset of the cohort over age 25 years limited further analysis of the impact of age on HRQOL at the various time points; this small group could relate to Sweden’s public health care system and school age screening resulting in surgical intervention for adolescent scoliosis. Another reason for the comparably low age in this study is that we specifically only included patients with idiopathic scoliosis with a juvenile or adolescent onset and treated as adults, and excluded adult degenerative spinal deformity. Whether treatment results in idiopathic and degenerative scoliosis differ may be an area of future studies. Nevertheless, the prospective, national register design of this study, meeting critical power calculations and reporting on the effect of surgery on two HRQOL outcome measures specifically in the adult surgically treated idiopathic scoliosis population at 2-year follow-up, represent strengths of this study and contribute to the literature in the discussion of evidence based treatment options.

## Conclusions

In conclusion, surgery has a positive effect on HRQOL outcomes, as reported by the EQ-5D index and SRS-22r subscore, at both 1- and 2-year follow-ups, potentially peaking at 1-year follow-up. The postoperative behavior of the different HRQOL outcome tools suggests the need for careful evaluation and interpretation both from a research and clinical perspective.

## Abbreviations

EQ-5D: EuroQol-5 dimensions
HRQOL: Health-related quality of life
SRS-22r: Scoliosis Research Society-22r questionnaire
SweSpine: The Swedish Spine register
